# Defect Analysis and Improvement Method of Eccentric Camshaft Forging by Vertical Upsetting Extrusion Forming

**DOI:** 10.3390/ma18071468

**Published:** 2025-03-26

**Authors:** Tao Wang, Hongxing Sun, Nan Hu, Dan Liu, Zhen Wang, Guanghui Liu, Chao Zhang, Hua Liu

**Affiliations:** 1 China Academy of Machinery Zhengzhou Research Institute of Mechanical Engineering, Co., Ltd., Zhengzhou 450001, China; 13623815363@163.com (T.W.); hunan@zrime.com.cn (N.H.); liudan@zrime.com.cn (D.L.); liugh@zrime.com.cn (G.L.); zhangchao@zrime.com.cn (C.Z.); 2 School of Mechanical Engineering, Xi’an Jiaotong University, Xi’an 710049, China; 3 School of Automation, Zhengzhou University of Aeronautics, Zhengzhou 450046, China; wangzhen@zua.edu.cn

**Keywords:** eccentric camshaft, upsetting extrusion forming, microstructure, metal streamline, crack

## Abstract

Eccentric camshaft components serve as critical elements in emergency pump systems for commercial vehicle steering mechanisms. To optimize material utilization efficiency, reduce production costs, and enhance manufacturing throughput, this investigation implemented a vertical upsetting extrusion forming methodology for camshaft forging production. Initial trials revealed defect formation in forged components. By analyzing the causes of the defects, an improved process method was developed to eliminate them. The chemical composition, macroscopic and microscopic morphologies of defects, forging process, and metal streamlines were analyzed and studied by means of a direct reading spectrometer, high-resolution camera, metallographic microscope, DEFORM finite element analysis software, and chemical etching. Findings indicate that the observed defects constitute forging-induced cracks, with subsequent normalizing heat treatment exacerbating decarburization phenomena in defect-adjacent microstructures. During the forging process of the forgings, the metal continuously extruded into the die cavity, and the inflowing metal pulled the dead zone metal downward, causing the flow lines aligned with the contour to bend into S-shaped metal streamlines. Cracks formed when the tensile stress in the dead zone metal exceeded the material’s critical tensile stress. An improved process was proposed: adopting a vertical upsetting extrusion forming method with a 40° diversion angle at the junction between the first step and the thin rod in the die cavity. Numerical simulations confirmed complete elimination of deformation dead zones in the optimized process. Experimental verification demonstrated crack-free forgings. Therefore, the eccentric camshafts formed by the initial process exhibited forging cracks, and the proposed improved method of vertical upsetting extrusion forming with a diversion angle effectively eliminated the forging cracks.

## 1. Introduction

**Figure 1:**
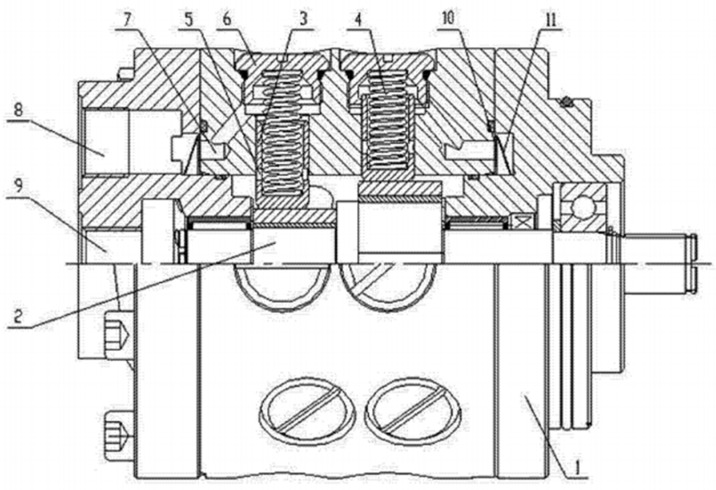
Structure diagram of directional pump: 1—pump body, 2—eccentric camshaft, 3—piston, 4—column spring, 5—oil suction hole, 6—piston hole, 7—oil outlet hole, 8—oil outlet pipe, 9—oil inlet pipe, 10—spring plate, 11—arc spring.

Commercial vehicle recirculating-ball steering systems predominantly employ hydraulic power assistance to manage substantial steering loads. A critical safety concern arises when primary hydraulic control systems fail, rendering mechanical steering insufficient for emergency maneuvers under dynamic driving conditions [1,2,3,4]. Regulatory mandates, including the European Commission Implementing Regulation (EU) 2021/535 and China’s GB 17675-2021, enforce stringent requirements for emergency steering functionality in heavy-duty vehicles (>12 t gross weight). These stipulate that, during power-assisted steering failure at speeds exceeding 10 km/h, driver hand force must remain below 450 N while achieving a 20 m radius turn within 6 s [5,6]. To address these requirements, an advanced emergency steering system was engineered as a redundant safeguard for commercial vehicles. This system integrates an emergency standby module into conventional hydraulic steering architectures, featuring autonomous activation via oil pressure feedback during primary system failure or engine shutdown [7,8,9,10]. The specific implementation method is to set the power take-off port on the gearbox, assemble the emergency pump, and arrange the emergency valve. During primary oil pump failure or engine stall, the emergency pump sustains hydraulic assistance using residual kinetic energy from vehicle motion, enabled by the emergency valve’s seamless flow-switching capability [11]. As the ultimate safeguard for steering integrity, the emergency pump’s operational reliability directly impacts vehicle safety. Figure 1 shows the structure diagram of an emergency pump, which has the advantage of stable flow [12]. The eccentric camshaft serves as the kinematic heart of emergency pump systems, where its precisely engineered rotational eccentricity drives plunger reciprocation to achieve controlled hydraulic fluid displacement. During operation, this critical component sustains complex torsional loading while maintaining dimensional stability under cyclic stress conditions. As the ultimate safety-critical element in vehicular steering architectures, rigorous manufacturing protocols must ensure complete elimination of metallurgical imperfections—surface discontinuities, microstructural anomalies, or stress concentrators—that could initiate premature fatigue failure.

The eccentric camshaft, as a type of stepped shaft part, can be manufactured through mechanical processing, forging and other methods. Ra Jo et al. [13] introduced a six-stage progressive rapid upset forging process tailored for producing a high-strength, one-piece input shaft with extended length and a remarkable material recovery rate. This process successfully manufactured an input shaft exhibiting high strength, hardness, torque capacity, and fatigue resistance. However, this methodology is primarily suited for the formation of axially symmetric stepped shaft forgings, leaving positioning and ejection challenges in eccentric shaft forgings at various stages to be addressed. A.N. Saquib et al. [14] conducted a finite element analysis on the cold forging process of a V8 engine camshaft. They evaluated the impact of incremental cam radius values on under filling and flash formation in the preform through numerical simulations, ultimately yielding an optimal preform geometry. This method entails three stages: preforging, final forging, and trimming, necessitating the coordination of multiple pieces of equipment. It is ideal for forming stepped shafts with numerous steps and intricate shapes. Sutao Han et al. [15] investigated the formation of stepped shafts using cross-wedge rolling technology. Through numerical simulations and experimental research, they discovered that employing convex-end billets could effectively mitigate defects in the concave centers of multi-stage shaft forgings. Cross-wedge rolling is particularly suitable for forming axisymmetric stepped shaft forgings. Tsu-Chin Tsao et al. [16] explored the design, modeling, and motion control of the non-circular turning process (NCTP) in camshaft machining. They designed and constructed an advanced NCTP turning test fixture based on state-of-the-art hardware and digital control to assess the feasibility of camshaft machining using NCTP technology. While the turning process offers low processing efficiency and material utilization, it excels in processing stepped shaft forgings with high precision and complex shapes, albeit at a relatively high cost for mass production. Through theoretical and experimental research, W.S. Weronski et al. [17] demonstrated the feasibility of open-die forging eccentric shafts on a three-slide forging press (TSFP). The use of tools with recesses in the lower clamping dies induced asymmetric material flow during upsetting, resulting in eccentric forming. However, this method lacks control over the external contour of the eccentricity and requires process allowances, leading to material loss. Iaroslav G. Zhbankov et al. [18] proposed various novel schemes for forging components like plates, shafts, and discs using free forging methods. For shaft parts, they found that forging rectangular cross-section workpieces using flat plates with stages was optimal, resulting in additional treatment at the billet center and a more uniform grain size distribution. However, open-die forgings suffer from poor shape accuracy, necessitating large subsequent machining allowances and exhibiting low material utilization efficiency. For non-symmetric stepped eccentric camshafts, existing manufacturing methods pose significant challenges and are relatively costly. Therefore, there is an urgent need to develop an efficient and cost-effective manufacturing process.

**Figure 2:**
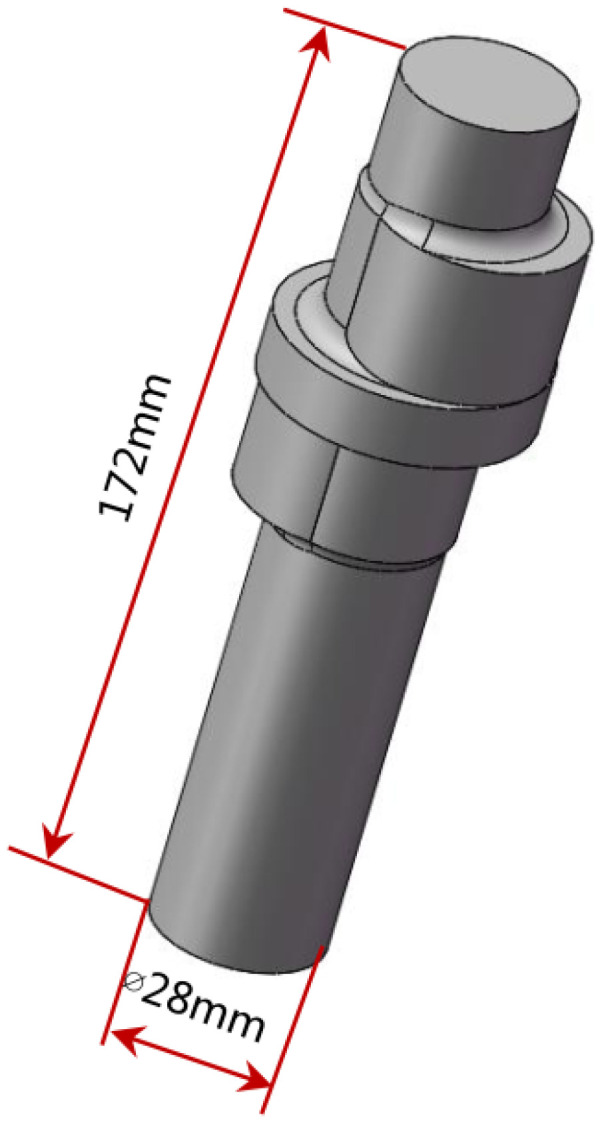
Three-dimensional diagram of eccentric shaft forgings.

**Figure 3:**
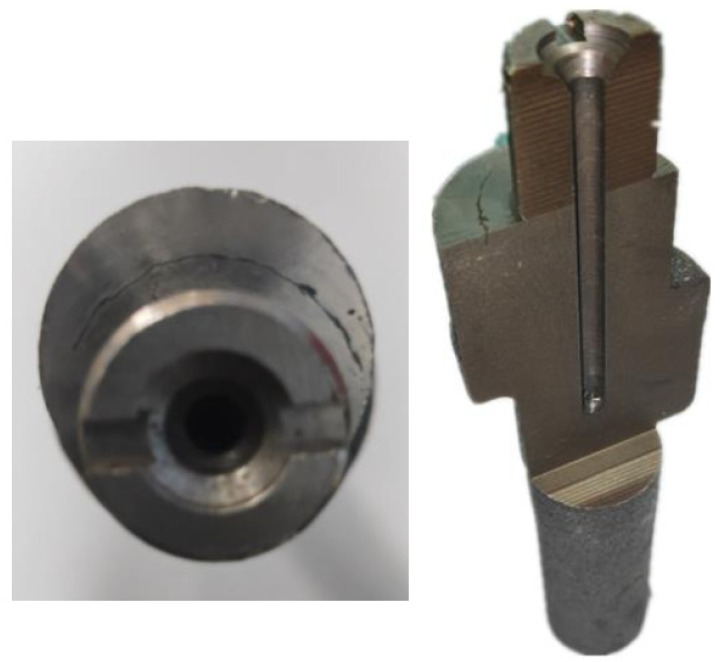
Macroscopic form of defects.

In this study, eccentric shaft forgings were successfully manufactured by a vertical single-direction upsetting extrusion forming process. As shown in Figure 2, the forging has two eccentric steps, with the first step and the second step being angled eccentric steps. There is a cylindrical flange between the two steps, which is coaxial with the thin rod portions at both ends of the forging. This innovative technique demonstrates multiple advantages: burr-free forming significantly improves material utilization. Localized bar heating achieves substantial energy savings. Single-step forming ensures high production efficiency. During machining operations, circumferential defects with approximately 145° angular distribution were observed at the first step after removing 2 mm from both the outer diameter allowance of the slender rod and the upper end face, as shown in Figure 3. These discontinuities, exhibiting measurable depth and radial alignment with the rod’s central axis, were preliminarily identified as either folding defects or cracks. Such flaws pose significant safety risks as potential initiation sites for catastrophic fracture during camshaft operation [19,20,21,22]. In this study, through a comprehensive analysis of the distribution of defects and the forging process, the root causes of defects were analyzed, and an effective improvement method was proposed.

## 2. Materials and Methods

### 2.1. Materials

The steel used for the eccentric camshaft is 20CrMnTiH. The chemical composition was detected with the aid of a spectrometer. The chemical composition analysis results of the eccentric camshaft forging are shown in Table 1. The test results and standard requirements are listed in the table. According to the composition requirements of 20CrMnTiH in GB/T3077-2015 [23], the chemical composition of the eccentric camshaft forging meets the standard requirements.

### 2.2. Initial Forming Process

**Figure 4:**
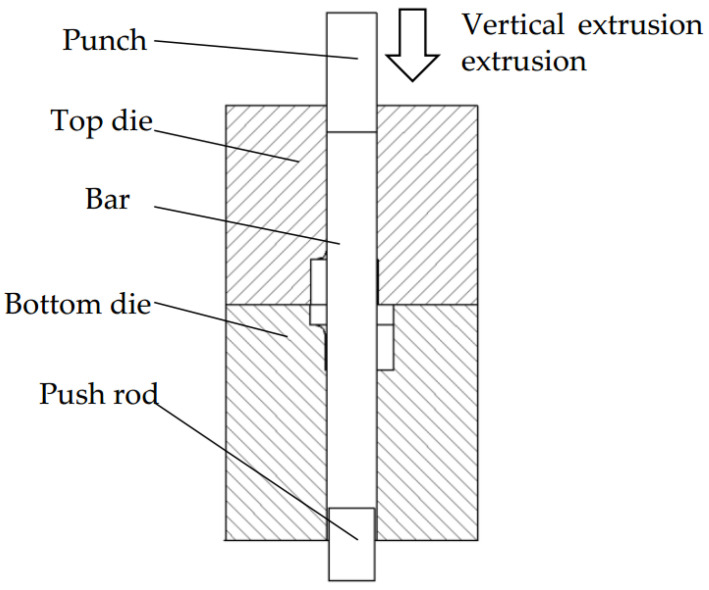
Vertical upsetting extrusion forming die diagram.

Vertical upsetting extrusion forming process for camshaft forgings: The bar is locally heated to 1050 °C, and then placed into the die. The punch gradually fills the die cavity with metal through unidirectional upsetting extrusion until the forging is formed, with a punch extrusion speed of 10 mm/s. The forming die is illustrated in Figure 4. After normalizing heat treatment, the formed forging achieves a homogeneous microstructure, significantly refined grain size, and complete elimination of residual forging stresses. Finally, the forging is ground to remove oxide scale.

### 2.3. Defect Analysis Method

Forgings in a normalized state were sliced and sampled by electrical discharge machining (EDM). The sampling position was perpendicular to the end face of the first eccentric step, and the sample thickness was 2 mm. High-resolution cameras are used to take photos of the sample, and the sampling depth is measured using the image software ImageJ-fiji. The samples in the normalized state and the forged state were cut along the axisymmetric centerline of the first eccentric step with defects, and the plastic forming part of the forging was locally sampled. The sample was a sheet with a thickness of 5mm. The samples were polished using diamond papers (#280 up to #1200) followed by diamond paste with a grain size of 0.25 μm. The polished mosaic sample was etched with a 4% nitric acid alcohol solution. The microstructure of the sample was observed with a Zeiss metallographic microscope. The sample was placed in a 10% nitric acid solution in a water bath for 20 min, and the corrosion forging streamline was observed. The forging process was simulated by DEFORM v11.0 software.

## 3. Results and Discussion

### 3.1. Macroscopic Morphological Analysis of Forging Defects

**Figure 5:**
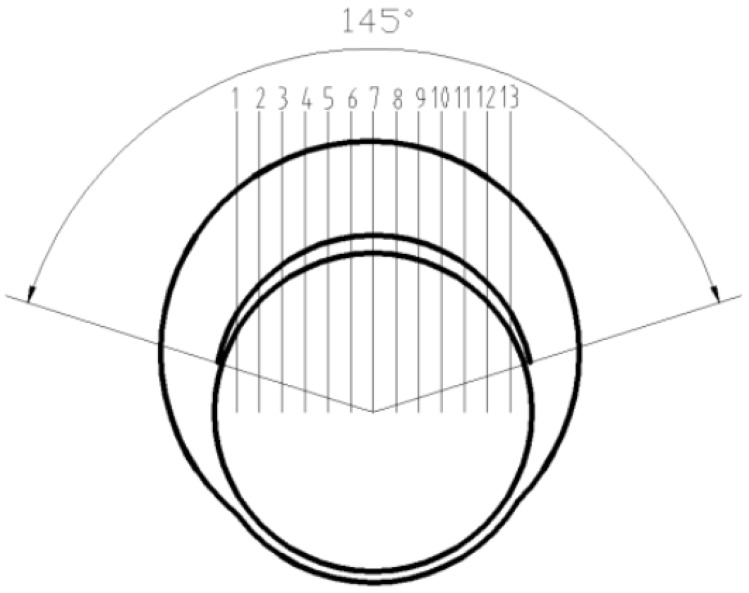
Slice sampling position.

**Figure 6:**
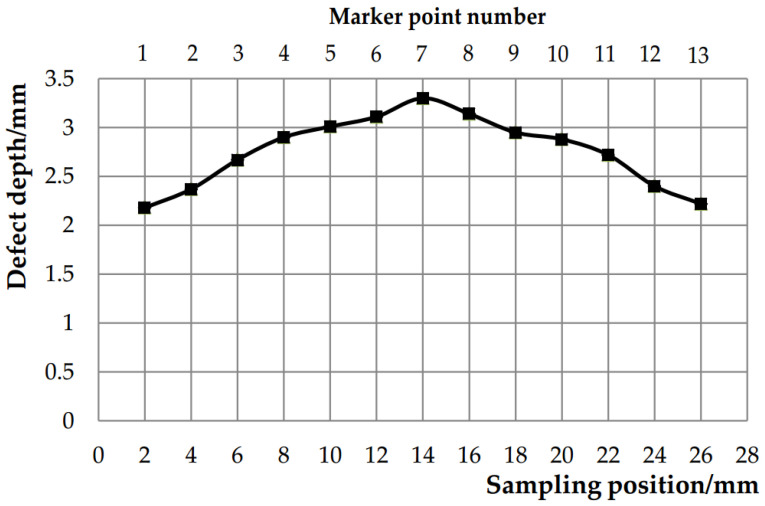
Marker point defect depth.

**Figure 7:**
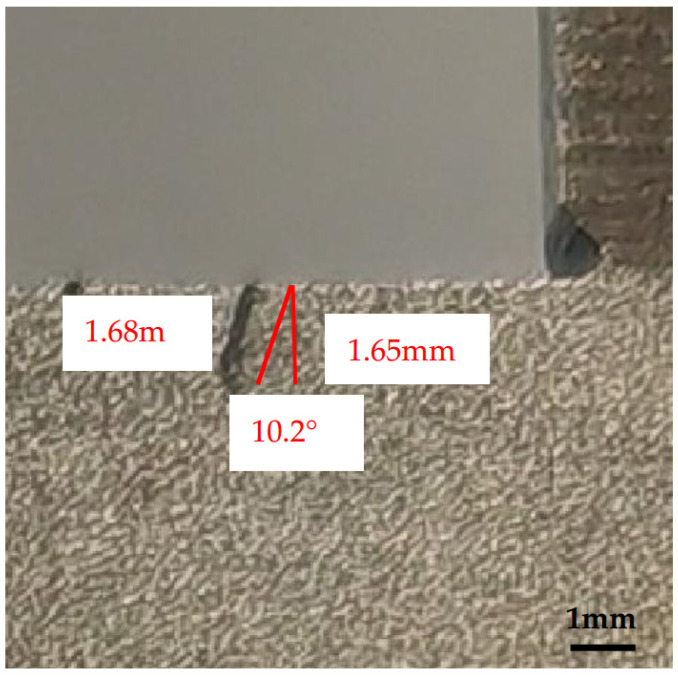
Marker point 7 cross-section defect morphology.

The forging defect manifests as an irregular semicircular depression on the end face of the first eccentric step, exhibiting significant depth as illustrated in Figure 5. To quantify defect characteristics, cross-sectional analysis was performed by sectioning the forging along the first step’s symmetry centerline. Twelve symmetrical 2 mm thick slices were prepared from both sides of the centerline (Figure 6). Each slice enabled depth measurement at thirteen predetermined markers perpendicular to the machined upper end face. Notably, the recorded depth measurements account for the 2 mm material removal from the first step’s upper surface during machining. The composite depth profile (Figure 6) reveals maximum defect penetration at Marker 7 along the eccentric shaft’s centerline, demonstrating an actual subsurface depth of 3.65 mm with a residual measured depth of 1.65 mm post-machining. Cross-sectional examination at Marker 7 (Figure 7) quantifies the defect geometry with precise dimensions: 1.68 mm in length and 1.65 mm in vertical depth relative to the machined surface. The defect exhibits a 10.2° angular deviation from the vertical axis normal to the upper end face.

### 3.2. Microstructure Analysis of Normalized and Forged Forgings

**Figure 8:**
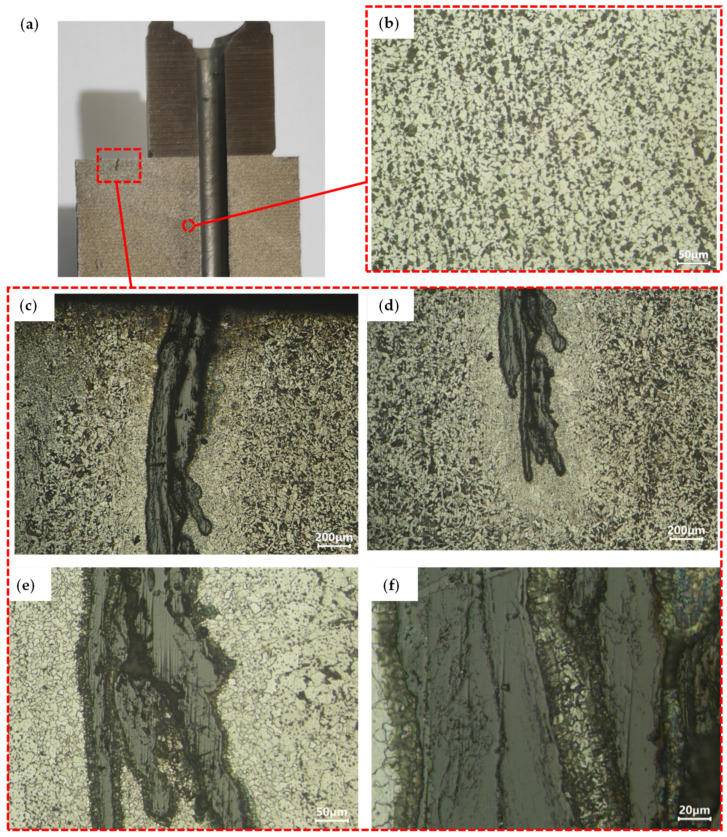
Microstructure analysis of normalized eccentric shaft: (**a**) Microstructure observation position, (**b**) The microstructure of the core, (**c**) The microstructure of the upper half of the defect, (**d**) Microstructure of the lower half of the defect, (**e**) Local amplification of 200 times of the microstructure at the tail of the defect, (**f**) Local amplification of 500 times of the microstructure at the tail of the defect.

Figure 8a presents the sampling location for metallographic examination of the normalized component. The core of the part’s microstructure (Figure 8b) demonstrates a homogeneous distribution of white ferrite and black pearlite with refined grain morphology, as shown in Figure 8b. Figure 8c,d illustrate the microstructural characteristics adjacent to the defect, revealing two notable phenomena: (1) dendritic branching at the defect terminus, and (2) extensive ferrite formation along the defect periphery. Quantitative microanalysis confirms progressive surface decarburization with increasing proximity to the defect interface. Notably, the microstructure transitions gradually to conventional ferrite–pearlite morphology with increasing distance from the defect zone (Figure 8c vs. Figure 8b). High-magnification imaging of the defect terminus reveals lamellar ferrite aggregates aligned parallel to the defect propagation axis as shown in Figure 8e,f. These elongated ferrite formations appear encapsulated within the primary defect structure. Given the implementation of isothermal normalization post-forging, the temporal formation mechanism (forging-induced vs. heat-treatment-related) requires further investigation. Comparative microstructural analysis of non-normalized counterparts from identical forging positions is needed to establish defect chronology.

**Figure 9:**
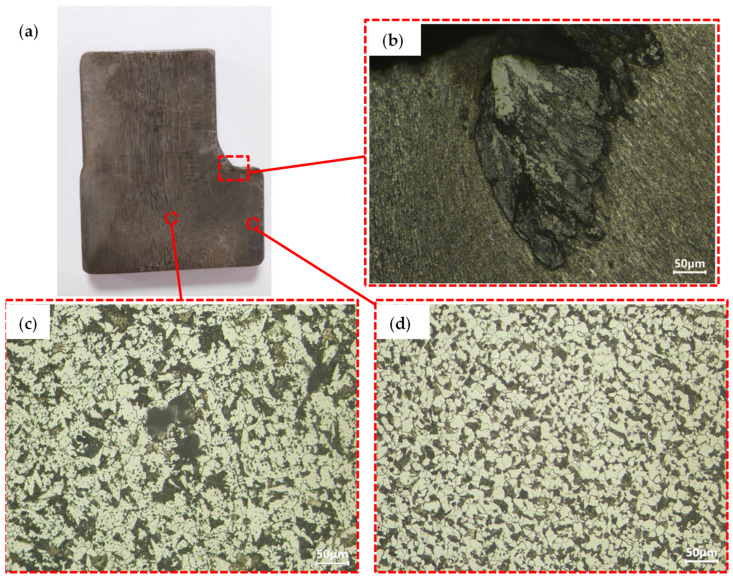
Microstructure analysis of eccentric shaft in forging state: (**a**) Microstructure observation position, (**b**) The microstructure of the surface defect location, (**c**) Microstructure of the core, (**d**) Microstructure of the Subsurface.

The metallographic observation site for the air-cooled forging is illustrated in Figure 9a. While surface defects appear in similar locations as observed in normalized components, their penetration depth is notably shallower in the forged specimen. Figure 9b presents the complex microstructure adjacent to the defect zone, revealing a heterogeneous structure that deviates from conventional ferrite–pearlite configurations [24,25,26]. This surface phenomenon can be attributed to differential cooling dynamics: the forged surface experiences rapid air cooling that promotes undercooled austenite transformation into ferrite and pearlite. However, residual heat from the forging’s high-temperature core subsequently conducts to the surface region, inducing tempering effects that create this distinctive composite microstructure. Notably absent are significant white-phase formations or pronounced decarburization patterns around the defect area. Cooling gradient effects are further demonstrated through metallographic analysis: the core region exhibits slower cooling-derived high-temperature transformation products—characteristic ferrite–pearlite structures, as shown in Figure 9c. The subsurface transition zone displays refined ferrite–pearlite morphology resulting from intermediate cooling rates between surface and core conditions, as shown in Figure 9d.

The analytical results conclusively demonstrate that the observed defects in forged components originate not from the normalizing heat treatment but arise during the forging formation process. Metallographic examination reveals that, during normalizing of as-forged components containing these imperfections, atmospheric exposure at defect interfaces initiates accelerated decarburization reactions. This mechanism accounts for the significantly greater depth of decarburization zones surrounding defects in normalized specimens when compared to their as-forged counterpart. Microstructural evidence further suggests that pre-existing forging defects function as stress concentration points during the normalizing process, resulting in further deepening of the defects.

### 3.3. Numerical Simulation Analysis of Eccentric Camshaft Forming

Based on the shape features and process characteristics of the camshaft, the bar model and die model were established using SolidWorks 2014 software. The mold structure is shown in Figure 3. These models were imported into the DEFORM v11.0 software in STL format. Boundary conditions were defined in DEFORM-3D following established methodologies from previous studies [27,28]. The approximate number of elements after solid mesh generation was 200,000. The material flow stress model was selected with 20CrMnTiH in DEFORM-3D. The deformation temperature in the numerical simulation was 1050 °C, with an isothermal deformation process and thermal conduction effects neglected. The punch extrusion step was set to 0.2 mm/step, and the punch extrusion speed was 10 mm/s. The object type of the workpiece was defined as a plastic body, and the mold was defined as a rigid body.

**Figure 10:**
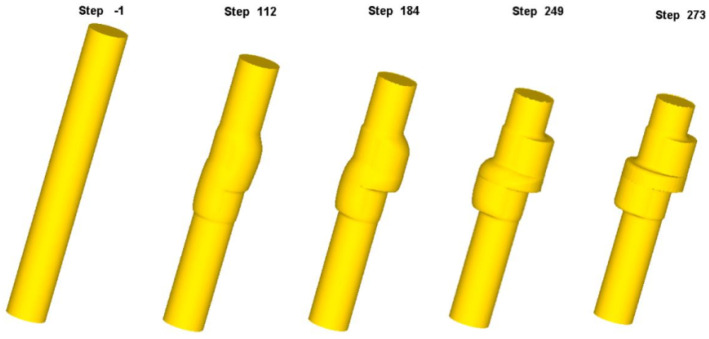
Numerical simulation of eccentric camshaft forming.

The numerical simulation process of eccentric camshaft forming is shown in Figure 10. As the punch continuously squeezed the upper end of the bar blank, the blank metal progressively flowed into the die cavity, filling the first step, the middle flange, and the second step. As more metal filled the mold cavity, the first step became completely filled at step 249. The last filling part of the first step was located at the sharp corner of the upper end face. At this stage, the middle flange side and the sharp corner of the lower end of the second step remained incompletely filled. The blank was continuously extruded into the die cavity, and the middle flange and the second step were fully filled, completing the shaping process. Throughout the forming sequence, no folding caused by metal reflux was observed.

**Figure 11:**
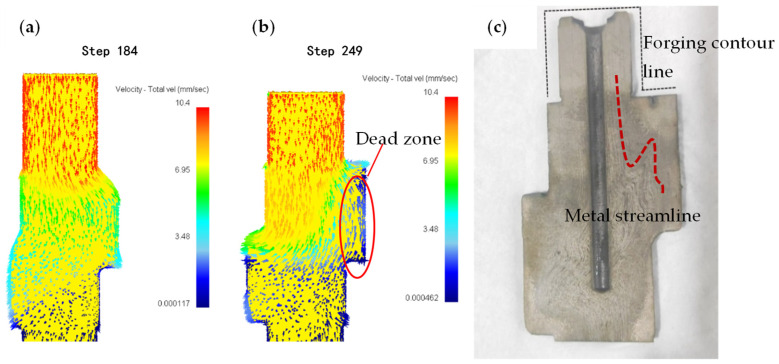
Metal flow analysis: (**a**) Step 184 simulation results, (**b**) Step 249 simulation results, (**c**) Experimental result of metal streamline.

Figure 11 presents comparative analyses of metal flow characteristics during eccentric camshaft forging, encompassing numerical simulations and experimental validation. The numerical simulation results are shown in Figure 11a,b. And the actual metal streamline detection results are shown in Figure 11c. The punch squeezed the bar metal, and the upper part of the bar metal rapidly flowed down into the die cavity. Upon entering the die cavity, the metal flow rate decreased, the flow direction changed, and the metal flowed in the direction of the minimum resistance, as shown in Figure 11a. When the first step became fully filled, a deformation dead zone was formed on the eccentric side of the first step, where metal flow essentially ceased. However, the metal in the upper part of the bar continued to squeeze into the die cavity to fill the remaining unfilled space of the middle flange and the second step. The metal flowing into the die cavity would also pull the metal located in the deformed dead zone at the first step, as shown in Figure 11b. The actual metal flow line of the forging also confirms the simulation results, as shown in Figure 11c. The metal streamline at the first step is S-shaped. After the first step was filled, the metal streamline should be distributed along the first step contour. When the metal continued to squeeze into the die cavity, the metal flowing into the metal would pull the metal in the dead zone downward, and the streamline distributed along the contour would be bent to form an S-shaped streamline. If the induced tensile stress in the dead zone exceeds the material’s critical tensile strength, forging cracks will initiate. Consequently, it is concluded that defects in the eccentric camshaft forging manifest as process-induced cracks during the forging process.

## 4. Defect Elimination Method

From the above analysis, the defect observed in the eccentric camshaft forging constitutes a typical forging crack. The crack initiation mechanism stems from the distinctive metal flow characteristics during the unidirectional upsetting extrusion forming process: the dynamic material flow generates significant tensile stresses within the deformation dead zone, exceeding the material’s critical fracture strength. These cracks initially form during the forging stage, subsequently serving as stress concentration sites during the normalizing heat treatment. The combined effects of thermal stress and phase transformation stress facilitate crack propagation and dimensional growth, ultimately manifesting as macroscopic surface defects.

**Figure 12:**
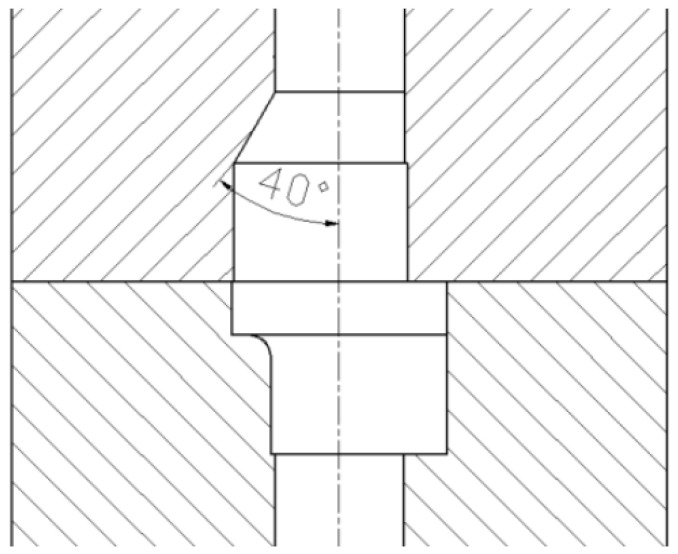
Diversion angle die diagram.

**Figure 13:**
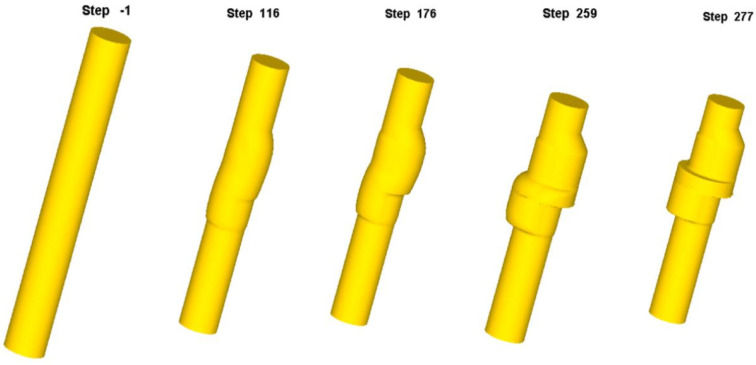
Numerical simulation of diversion angle method forming.

**Figure 14:**
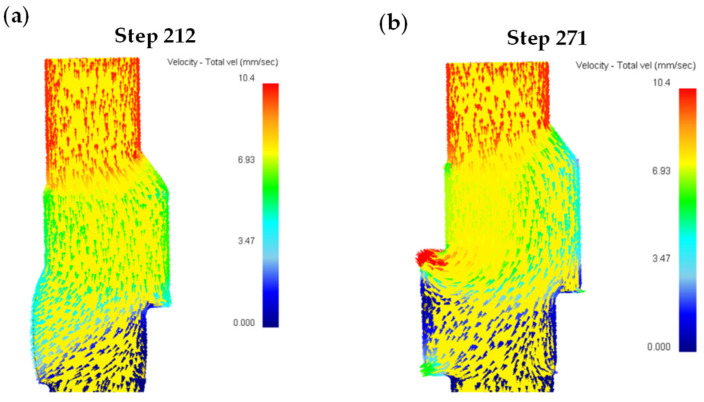
Metal flow analysis of diversion angle method: (**a**) Step 212 simulation results, (**b**) Step 271 simulation results.

Based on the systematic analysis of crack formation mechanisms, an improved forming scheme was proposed. The unidirectional upsetting extrusion forming method was adopted, with a 40° diversion angle set at the junction of the first step and the thin rod section in the die cavity, as shown in Figure 12. The numerical simulation model incorporated the improved die structure. Since the forging included the diversion angle, the length of the bar stock was increased accordingly. The punch extruded the bar blank at a speed of 10 mm/s, while other simulation parameters remained unchanged. The numerical simulation of the forming process is shown in Figure 13. During the forming process, the blank metal was unidirectionally extruded into the die cavity, with the first step being filled first, followed by the gradual filling of the second step and the middle flange part as the metal continued to extrude. After the first step was filled, no deformation dead zone formed at its sharp corner, as shown in Figure 14a. As the blank metal was further extruded, the metal in the first step flowed downward as a whole and moved into the unfilled areas of the middle flange and the second step, as shown in Figure 14b.

**Figure 15:**
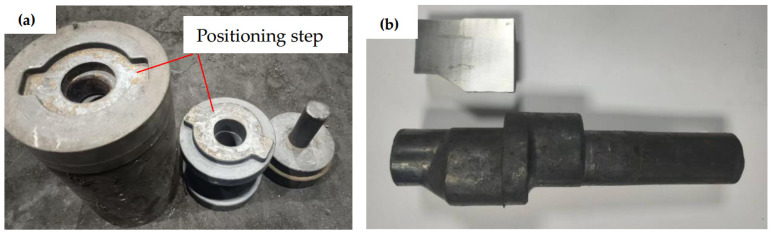
Diversion angle method experiment; (**a**) Diversion angle die, (**b**) Eccentric camshaft forging.

The experimental trials were performed using a 3.15 MN hydraulic press system. Figure 15a illustrates the custom-designed forming dies, which incorporated a precision positioning step on their end faces to ensure proper die alignment during operation. As demonstrated in Figure 15b, the resultant eccentric camshaft forgings exhibited complete die filling characteristics with no observable crack formation. These results confirm that the innovative integration of unidirectional upsetting extrusion forming methodology with strategically designed diversion angles in the die structure successfully addresses the persistent crack defect challenges associated with eccentric camshaft forging processes.

## 5. Conclusions

This study investigates defects in the vertical upsetting extrusion forming process of eccentric camshaft forgings. By analyzing the macroscopic and microscopic morphology of the defects, the defect formation mechanism was identified. The metal flow in the forming process was analyzed by the numerical simulation method, and the simulation results were compared with the actual metal streamline of the forging to determine the essence of the defect. According to the mechanism of defect generation, a method to eliminate defects has been proposed and validated through numerical simulations and experimental methods. The conclusions are as follows:

(1) Defects in vertical upsetting extrusion camshaft forgings originate during the forging process. The subsequent normalizing process exacerbates decarburization near the defects. The defects function as stress concentration points during the normalizing process, resulting in further deepening of the defects.
(2) The defect in vertical upsetting extrusion camshafts is cracking. During forming, the first step of the forging is initially filled, creating a deformation dead zone. At this stage, metal flow lines at the first step align with its contour. As metal continues to extrude into the die cavity, the inflowing metal pulls the dead zone metal downward, bending the contour-aligned flow lines into an S-shaped pattern. Cracks form when the tensile stress in the dead zone exceeds the material’s critical tensile stress.
(3) To address crack formation, an optimized forming strategy was developed: a unidirectional upsetting extrusion method with a 40° diversion angle incorporated at the junction between the first step and the thin rod in the die cavity. Numerical simulations confirmed that no deformation dead zone formed at the first step after unidirectional extrusion of the billet into the die cavity. Post-filling of the first step, the metal in this region flowed downward uniformly, redistributing into unfilled areas of the middle flange and second step. Experimental validation of the diversion-angle-integrated unidirectional forming method demonstrated crack-free forgings, proving the effectiveness of the improved approach.

The improved process for forming eccentric camshaft forgings incorporates a diversion angle structure, which results in a small amount of raw material waste. The forthcoming research endeavors to develop a new double-direction upsetting extrusion process for forming camshaft forgings. This process allows the dies to simultaneously press on both ends of the billet, promoting short-distance metal flow to fill the die cavity, thereby achieving precise forging of the component and improving material utilization.

## Figures and Tables

**Table 1:** Eccentric camshaft forging elements content and standard range of 20CrMnTiH elements (wt%).

Element	C	Si	Mn	P	S	Cr	Cu	Ti
**Results**	0.19	0.23	0.96	0.01	0.01	1.05	0.007	0.08
**Standard Value (GB/T3077-2015)**	0.17~0.23	0.17~0.37	0.80~1.10	≤0.035	≤0.035	1.00~1.30	≤0.035	0.04~0.10

## Data Availability

The data presented in this study are available upon request from the corresponding author. The data are not publicly available due to the project’s confidentiality requirements.
